# The Database of Genomic Variants: a curated collection of structural variation in the human genome

**DOI:** 10.1093/nar/gkt958

**Published:** 2013-10-29

**Authors:** Jeffrey R. MacDonald, Robert Ziman, Ryan K. C. Yuen, Lars Feuk, Stephen W. Scherer

**Affiliations:** ^1^The Centre for Applied Genomics, Peter Gilgan Centre for Research and Learning, The Hospital for Sick Children, 686 Bay Street, Toronto, Ontario M5G 0A4, Canada, ^2^Department of Immunology, Genetics and Pathology, Science for Life Laboratory, Uppsala University, Uppsala SE-751 08, Sweden and ^3^Department of Molecular Genetics, University of Toronto, Toronto, Ontario M5S 1A8, Canada

## Abstract

Over the past decade, the Database of Genomic Variants (DGV; http://dgv.tcag.ca/) has provided a publicly accessible, comprehensive curated catalogue of structural variation (SV) found in the genomes of control individuals from worldwide populations. Here, we describe updates and new features, which have expanded the utility of DGV for both the basic research and clinical diagnostic communities. The current version of DGV consists of 55 published studies, comprising >2.5 million entries identified in >22 300 genomes. Studies included in DGV are selected from the accessioned data sets in the archival SV databases dbVar (NCBI) and DGVa (EBI), and then further curated for accuracy and validity. The core visualization tool (gbrowse) has been upgraded with additional functions to facilitate data analysis and comparison, and a new query tool has been developed to provide flexible and interactive access to the data. The content from DGV is regularly incorporated into other large-scale genome reference databases and represents a standard data resource for new product and database development, in particular for copy number variation testing in clinical labs. The accurate cataloguing of variants in DGV will continue to enable medical genetics and genome sequencing research.

## INTRODUCTION

Structural variation (SV) refers to the balanced or unbalanced changes in DNA content, which include both cytogenetically visible, submicroscopic and even smaller sequence-level variants. In the past 10 years, new genomic technologies of increasing resolution have revealed SV to be ubiquitous in all human DNA and often involved in disease (1), with unbalanced alterations of DNA, called copy number variations (CNVs) or smaller insertion/deletion (indel) events encompassing an order of magnitude more nucleotides than even single nucleotide polymorphisms (SNPs) (2). DNA variations that are balanced in nature, such as inversions and translocations, are less common in the human genome, but can also be important in chromosomal evolution and disease (3).

**Figure 1.**
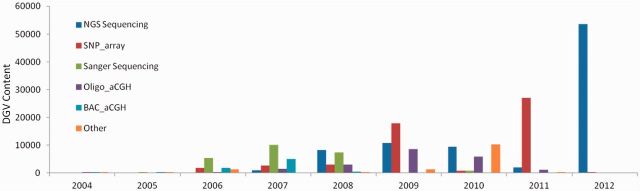
Content of the DGV. Increase in variants reported in DGV since inception, highlighting the recent transition towards NGS-based approaches for variant discovery (numbers based on year of publication).

The Database of Genomic Variants (DGV) was launched following the publication of the inaugural CNV articles that described the genome-wide prevalence of CNV in the genomes of healthy, clinically unaffected individuals (4,5). In its early iteration, the database comprised SV data from a few hundred individuals representing ∼1000 CNVs and some inversions (6). DGV has now expanded to encompass information from 55 studies with >2.5 million entries. The majority of the early studies in DGV were generated from low-resolution microarrays on a limited number of samples, which often had both high false-positive and false-negative rates (7). Several of those initial studies have now been removed from DGV as part of the ongoing curation process. Currently, higher resolution microarrays (8,9), and data from individual genome sequences, produced by massively parallel next-generation sequencing (NGS) (10,11), have begun to populate DGV, which significantly improve the accuracy of the curated SV catalogue (Figure 1) (12).

DGV aims to catalogue the highest quality SV described in the literature in a format accessible to medical geneticists and molecular biologists alike. Both researchers and clinicians use the data regularly either directly at the website, through tracks displayed at publicly accessible genome browsers including UCSC (13) and Ensembl (14) or through multiple commercial software tools (CytoSure, BlueFuse Multi, ChAS). Here, we describe the redevelopment of DGV, which has been motivated by an expanded number of disciplines using SV data for their genomics analyses.

## COLLABORATION/CONTENT AQUISITION/REPORTING

Recognition of the growing importance of SV in disease studies, necessitate the development of a long-term and stable archive of SV data. In 2008, a collaboration with DGVa (http://www.ebi.ac.uk/dgva) and dbVar (www.ncbi.nih.gov/dbvar) was established to create an archive, which allowed for the implementation of standardized terminology and assignment of formal accession numbers ensuring seamless access to these data (12). A pipeline was developed to exchange data between the DGVa and dbVar archives (15), and from the archives all data sets describing SV in healthy human control samples are sent to DGV for curation, interpretation and display (Supplementary Figure S1). This arrangement ensures a standardized set of terms and values are used to describe the various attributes, allowing DGV curators to consistently and effectively record and store this data. This allows users to effectively compare data across studies and across samples as each entry has been recorded in a consistent and well-defined manner. With the implementation of a direct submission template at DGVa/dbVar, DGV no longer accepts direct submissions, but instead obtains studies directly from DGVa (Supplementary Figure S1). Authors are encouraged to submit their raw data to the appropriate archive, either Gene Expression Omnibus (16) or Array Express (17) and processed variant calls to DGVa or dbVar. Provided the study passes curation and quality control, it will be selected for inclusion and display in DGV. The change in DGV’s data acquisition led to the implementation of new DGV accessions. Supporting structural variant calls (ssv), representing the underlying sample level or algorithm level records from a study are assigned an nssv (NCBI; dbVar) or essv (EBI; DGVa) accession. Supporting structural variant (ssv) calls represent a variant identified in a single sample from a single experiment. Studies that have analysed the same sample or set of samples on different platforms or using different tools/algorithms may therefore have multiple records for a single sample. Variant calls are summarized and a variant region is generated based on the specific assertion method (15). Variant regions are assigned an nsv (NCBI; dbVar) or esv (EBI; DGVa) accession.

To accurately represent the variant region and reduce the complexity associated with complex regions, an additional step is performed by DGV while processing variant regions within a single study. A DGV merged variant is created if there are a number of overlapping variant regions that are almost identical, but may be slightly different due to the inherent variability between experiments. If there are clusters of variants within a single study, which share at least 70% reciprocal overlap in size and location, these will be merged and an accession record that has our internal ‘dgv’ prefixed identifier will be provided.

## DATA CURATION/PROCESSING

The data available in the literature is derived from a multitude of experimental approaches and methodologies. All studies are carefully evaluated and curated to ensure only high-quality data are included in the database. A number of steps are performed to assess the data, identify and remove entries that may represent false positives (Supplementary Figure S1). Following the initial curation, an automated pipeline was developed to assess each individual variant and each study. Filters include, but are not limited to, removal of (i) variants erroneously mapped to the mitochondrial genome or on the Y chromosome in female samples; (ii) variants <50 bp (already well represented in dbSNP) (18), and CNVs larger than 3 Mb and inversions larger than 10 Mb, (iii) variants coinciding with gaps in the reference assembly, (iv) variants reported as causative for genomic disorders in DECIPHER (19) and (v) overlapping variants in the same individuals that cannot theoretically overlap (e.g. an inversion within a deletion on the X chromosome in a male). These filters currently impact 43 different studies (Supplementary Table S1), with the majority of the excluded variants falling below our size threshold. These filters are in place both to remove erroneous variants from data sets, but also to provide an overview of the quality of the data set as a whole, which may lead to the decision to exclude the entire study from DGV.

## DATA CONTENT

As new SV studies are generated or published, they are assessed for inclusion in the database. Over time, older studies may be removed or retired if the content is no longer the most accurate description of SV in those populations. In many cases, similar samples will be analysed on newer higher-resolution platforms and have provided superior representation of the actual biological variant in the region. The number of variants included in the database has increased rapidly throughout the years driven primarily by studies using NGS approaches for detecting SV (Figure 1). The most recent update to DGV includes 55 studies representing >2.5 million structural variants corresponding to 202 431 variant regions, which includes 1149 inversions (Table 1). Studies are derived from microarrays and sequencing, with four primary types of analysis: (i) array-based comparative genomic hybridization and comparative intensity analysis (SNP/CNV arrays); (ii) statistical analysis of SNP array data for deletion detection; (iii) clone end sequencing mapping; and (iv) sequence trace mapping. In the latest release of the database, 44% of the variants come from microarray studies and the remaining variants were identified in sequencing studies (53%), and other targeted approaches including FISH/PCR and Optical Mapping (3%). The size of CNVs ranges from 50 bp to 3 Mb, with a significant drop of variant numbers in 50 bp to 1 kb range (Supplementary Figure S2). This is primarily due to the inability of microarrays to detect small-scale CNVs. We anticipate the record of small-scale CNVs will continue to grow with the increased use of NGS. Sequence ontology terms have been used by DGVa and dbVar to describe the types of genomic SV included in the database (20). Although numerous types of SV are included, the database is enriched for deletions and copy number losses (70%), while copy number gains, duplications and insertions comprise ∼25% of the SV entries. Although there are few inversions and complex variants represented, this remains a unique and important class of SV catalogued in DGV (Table 1).

**Table 1.** DGV content

Database content	Number of entries
Studies	55
Unique samples	14 316
Variant regions	202 431
Deletion	77 268
Duplication	668
Loss	64 185
Gain	24 891
Gain + loss	3850
Insertion	24 140
Inversion	1149
Complex	4090
Unknown	2189
Variant calls	2 393 718
CNV	2 391 408
Inversion	2310
Filtered variants	3 900 253

An overall summary of the number of studies and samples reported in the database (July 2013 update, mapped to GRCh37 assembly). Individual variant types are reported highlighting the distribution of SV content in the database.

Many studies have used common/universal sets of control subjects (HapMap, HGDP, 1000G), but there is also a large number of unique cohorts, which increases the geographic representation of samples for comparison (Supplementary Figure S3). A total of 22 255 samples have been assayed across all the studies in the database representing a non-redundant total of 14 316 individuals. There is approximately equal representation of both male and female samples (53 and 47%, respectively), and they are derived from ∼44 different populations. The identification of variants on the Y chromosome is underrepresented owing to a number of factors (primarily technical). For example, complex palindromes, highly repetitive and GC rich content provide difficulty in targeting and interpreting regions on the Y chromosome.

## DATA PRESENTATION/ACCESS

SV data are made available in multiple formats providing graphical- (gbrowse), tabular- (query tool) and text-based formats (downloads) (Table 2).

**Table 2.** Overview of novel features incorporated in DGV

New tools/features	Categories	Description
Gbrowse	Navigation	Click and drag zoom capabilities on chromosome and/or position bar.
	Filter	Option to display only selected entries for DGV structural variant data.
	Export	Option to save data from DGV and annotation tracks to a text file for the region, chromosome or whole genome.
	Annotations	Additional relevant annotations including ISCA and DECIPHER consented patient data.
Query tool	Study	Information on each individual study in DGV.
	Variant	Complete list of all structural variants with details on mapping location, samples and the study of origin.
	Sample	Details on the identifier, gender, ethnicity and source of samples used in each study.
	Method	Description of discovery and validation methods used for each study.
	Platform	The name of the platform used in each experiment with links to GEO and Array Express.
	Analysis	Individual tools, algorithms and approaches used with associated descriptions.
	Export Options	Allows users to save output as csv, excel or PDF file.
	Filter Options	Can apply multiple search options across all fields in the database.
Variant details page	Allele State	Identifies if variant is heterozygous or homozygous.
	Allele Origin	Identifies if a variant is *de novo* or inherited.
	Copy Number	Reporting the absolute number of copies for a variant call.
	Allele length	The length of insertion sequences is listed when available.
	Probe number	The number of probes reported for an individual variant call.
	Method	Description of discovery and validation methods used for each study.
	Analysis	Individual tools, algorithms and approaches used to identify a variant.
	Platform	The name of the platform used in each experiment.
Accessions	nsv	NCBI structural variant (variant region).
	nssv	NCBI ssv (variant call).
	esv	EBI structural variant (variant region).
	essv	EBI ssv (variant call).
	dgv	DGV merged variant; generated if two or more variant regions share >70% reciprocal overlap within a study.

Improvements in the number of options for navigation and display (gbrowse) are outlined in addition to an overview of the content provided in the relevant tables (query tool). An increased number of attributes have been defined and reported (where applicable) and are outlined with details on the new SV accessions.

**Figure 2.**
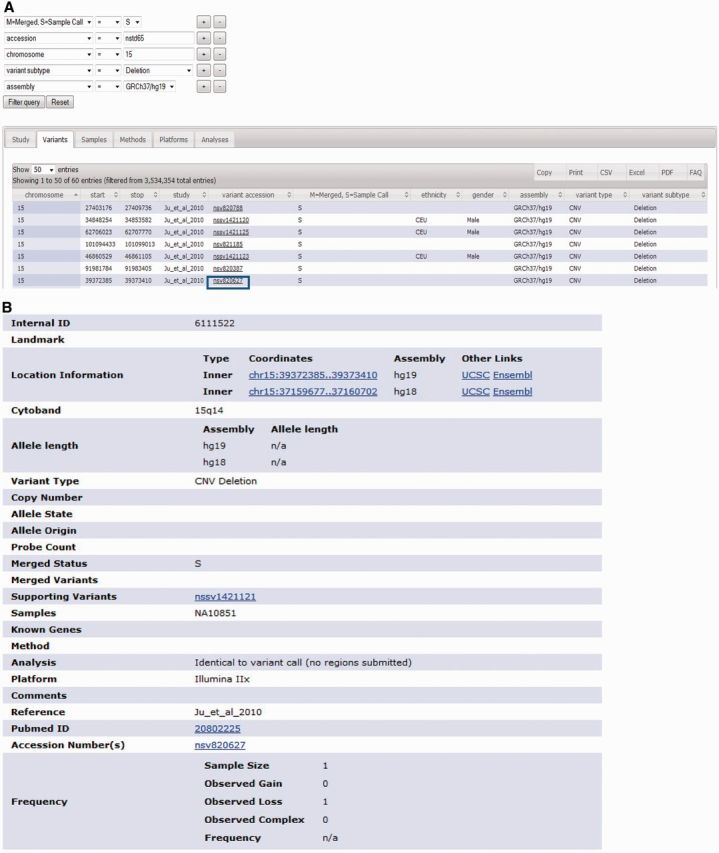

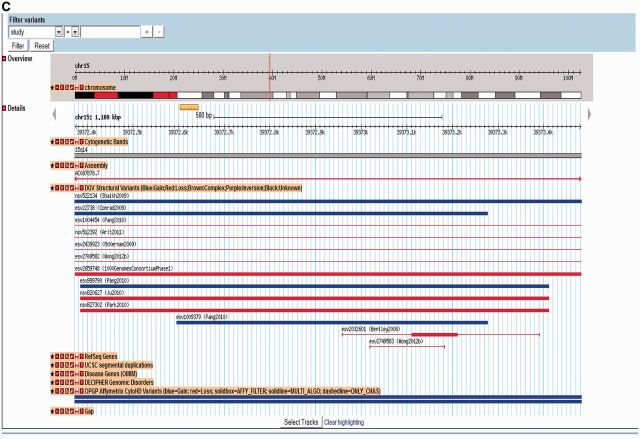
Functionality and navigation options for accessing entries in DGV. (**A**) An example of search options available in the DGV query tool, which identify sample level deletions in study nstd65 mapped to the GRCh37 assembly. (**B**) Links for each variant in the query tool result, allow for navigation to the variant details page, which includes a summary of all available attributes. (**C**) Links from the variant details page provide access to the genome browser to allow for evaluation of selected variants in their respective genomic region.

The genome browser is a graphical user interface, which uses the GMOD/Gbrowse (21) platform. SV data are displayed as a track and is subdivided to represent the variant regions and also the underlying sample level/supporting level variant calls. Additional annotations are displayed to allow for interpretation of the variation data in their genomic context (Figure 2). These include standard annotation tracks such as RefSeq (18) and OMIM genes (http://omim.org), segmental duplications (22), array probe files and a number of clinically relevant variant regions. These include the DECIPHER genomic disorders and consented patient data (19), and data sets from the ISCA consortium (23,24). Filtering options have been developed allowing for customized views of DGV data based on a selected number of options.

A query tool has been developed representing a searchable set of interrelated tables, which contain all the underlying information in the database. Options to search and filter the information within or across studies are also now possible (Figure 2), providing the option to customize the output based on a number of terms and attributes (Table 2). Information has been organized by various categories with relevant information provided on each individual study, details on samples analysed, the variants that were described and tables outlining the methods, platforms and analyses performed in each study (Table 2).

Data are exported and provided on the Downloads page and contain a copy of all the information contained in the database with variants mapped to multiple assemblies (NCBI36/hg18 and GRCh37/hg19 where applicable). This allows for fast, complete and easy access to the data. These are organized by release date, and updates are included alongside archived copies of earlier versions. These data are accessed by multiple users and are the primary site for distributing the content to other genome databases including UCSC (13) and GeneCards (http://www.genecards.org/) and commercial vendors who use the data for both product development and as an annotation track in various software packages and analysis suites.

## DATABASE STRUCTURE

To manage, host and display increasingly complex and numerous entries, the database was redeveloped to create a robust and scalable platform. The underlying data model has been provided (http://dgv.tcag.ca/dgv/app/index.html) and details on the database model, tools and pipelines are described in the Supplementary Materials.

## FUTURE DIRECTIONS

Considering the large number of variants stored and in the database, and the large fraction of the genome covered by SV, a more rigorously curated reference is now required for the data to have even more utility. With high-resolution microarrays and sequence-based annotation, SV data are of sufficient quality to develop such a resource. The future development of this new data track in DGV, the ‘Gold Standard of SV (or GSSV)', will be essential for accurate assessment of new technologies, annotating SV in genome assemblies (both reference assemblies and personal genome assemblies) and more precise clinical microarray comparisons. In our first GSSV release, we will initially build clusters of CNVs from the selected data sets. Each cluster may contain a single variant or many variants. Within each cluster, variants will be compared based on size (reciprocal overlap). Variants, which overlap and are of similar size and type may represent the same underlying variation, and would be manually curated. Other (non-CNV) SVs with sequenced breakpoints (e.g. inversions) will be added to the GSSV, which will be recompiled each time the DGV is updated. All underlying data will remain in DGV for reference. The goal of the GSSV track is to provide the users of the database with the best possible interpretation of existing data in terms of the location, frequency and breakpoint resolution.

## DISCUSSION

Since the inception of DGV ∼10 years ago, there has been a tremendous advancement in the technologies and informatics tools available to detect SV. The sensitivity and specificity of many early-generation SV-detection technologies was low and entries in DGV may be incorrect, or carry imprecise boundary coordinates or frequencies (7,25). Although many older studies have now been removed, and the user is given a choice to display only data from higher-resolution platforms, the use of DGV still requires a basic understanding of SV and how the field has developed to accurately interpret the data. The rapid uptake of microarray testing, and more recently, exome or whole-genome sequencing, in molecular diagnostic laboratories, is demanding that DGV continually refine its data content and database structure. As research and clinical endeavors expand, we anticipate the preponderance of new variants that will need further characterization, will be rare in nature and often unique to families or individuals. As discussed, DGV is prepared to meet these challenges and continue to facilitate the needs of the SV research community.

## SUPPLEMENTARY DATA

Supplementary Data are available at NAR Online.

## FUNDING

University of Toronto McLaughlin Centre; Ontario Genomics Institute/Genome Canada; Canada Foundation for Innovation; Canadian Institutes of Health Research (CIHR); The Centre for Applied Genomics and the Hospital for Sick Children Foundation. The GlaxoSmithKline-CIHR Chair in Genome Sciences at the University of Toronto and the Hospital for Sick Children (to S.W.S.). Funding for open access charge: Genome Canada Grant.

*Conflict of interest statement*. None declared.
